# Dilated Cardiomyopathy Revealing Cushing Disease: A Case Report and Literature Review

**DOI:** 10.1097/MD.0000000000002011

**Published:** 2015-11-20

**Authors:** Lucien Marchand, Bérénice Segrestin, Marion Lapoirie, Véronique Favrel, Julie Dementhon, Emmanuel Jouanneau, Gérald Raverot

**Affiliations:** From the Department of Endocrinology, Groupement Hospitalier Est, Hospices Civils de Lyon, Bron, France (LM, BS, ML, GR); Department of Radiotherapy, Centre Hospitalier de Lyon Sud, Hospices Civils de Lyon, Pierre-Bénite, France (VF); Department of Cardiology, Tonkin clinic, Villeurbanne, France (JD); Department of Neurosurgery, Groupement Hospitalier Est, Hospices Civils de Lyon, Bron, France (EJ); and INSERM U1028, CNRS UMR5292, Lyon Neuroscience Research Center, Neuro-Oncology and Neuro-Inflammation Team, Lyon, France (EJ, GR).

## Abstract

Cardiovascular impairments are frequent in Cushing's syndrome and the hypercortisolism can result in cardiac structural and functional changes that lead in rare cases to dilated cardiomyopathy (DCM). Such cardiac impairment may be reversible in response to a eucortisolaemic state.

A 43-year-old man with a medical past of hypertension and history of smoking presented to the emergency department with global heart failure. Coronary angiography showed a significant stenosis of a marginal branch and cardiac MRI revealed a nonischemic DCM. The left ventricular ejection fraction (LVEF) was estimated as 28% to 30%. Clinicobiological features and pituitary imaging pointed toward Cushing's disease and administration of adrenolytic drugs (metyrapone and ketoconazole) was initiated. Despite the normalization of cortisol which had been achieved 2 months later, the patient presented an acute heart failure. A massive mitral regurgitation secondary to posterior papillary muscle rupture was diagnosed as a complication of the occlusion of the marginal branch. After 6 months of optimal pharmacological treatment for systolic heart failure, as well as treatment with inhibitors of steroidogenesis, there was no improvement of LVEF. The percutaneous mitral valve was therefore repaired and a defibrillator implanted. The severity of heart failure contraindicated pituitary surgery and the patient was instead treated by stereotaxic radiotherapy.

This is the first case reporting a Cushing's syndrome DCM without improvement of LVEF despite normalization of serum cortisol levels.

## INTRODUCTION

Endogenous Cushing's syndrome may be caused by overproduction of adrenocorticotropic hormone (ACTH) (Cushing's disease in most cases) or by an independent high cortisol secretion from the adrenal cortex.^1^ Cardiovascular impairments are frequent, due to hypertension, hyperglycemia, hypercholesterolemia, weight gain, and a prothrombotic state. Hypercortisolism can also cause cardiac structural and functional changes^2–5^: hypertrophy, concentric remodeling, fibrosis, biventricular and left atrial systolic dysfunction, and diastolic dysfunction, leading in rare cases to dilated cardiomyopathy (DCM).

We present here the case of a man with DCM related to Cushing's disease (CD).

## CASE REPORT

A 43-year-old man with a medical history of hypertension for 8 years and history of smoking, presented to the emergency department with dyspnea. He was under treatment with amlodipine at a dose of 5 mg/day. Physical examination, blood tests, and chest imaging revealed global heart failure. Coronary angiography revealed an isolated significant stenosis in the marginal branch of the circumflex artery. Transthoracic echography showed a globally dilated heart (left ventricular end-diastolic diameter: 64 mm), a mild left ventricular hypertrophy, and a global hypokinesia with a more prominent posterolateral wall hypokinesis (probably explained by the stenosis of the marginal branch). Left ventricular ejection fraction (LVEF) was calculated as 28% to 30%. No significant valvular heart disease, no sign of pulmonary hypertension, and no pericardial effusion were noted. Cardiac magnetic resonance imaging (MRI) also showed a DCM, with no sign of necrosis. The patient reported no excessive alcohol consumption and investigations for usual infective, autoimmune and infiltrative causes of cardiomyopathy were negative.

**FIGURE 1:**
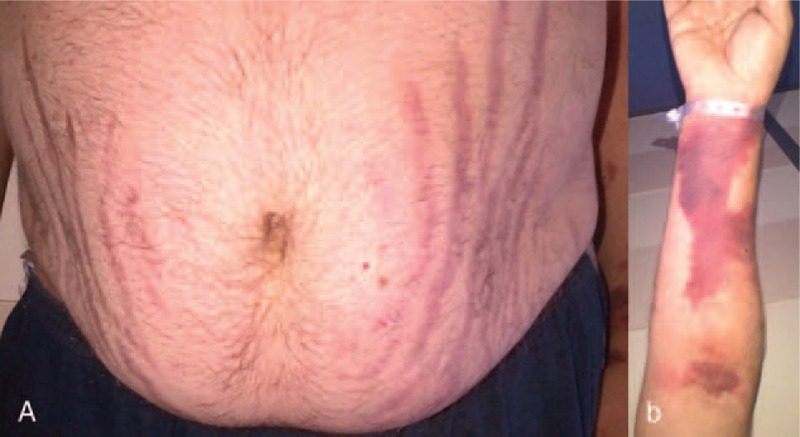
Reddish purple striae (A) and bruising (B) in a context of Cushing's syndrome.

**FIGURE 2:**
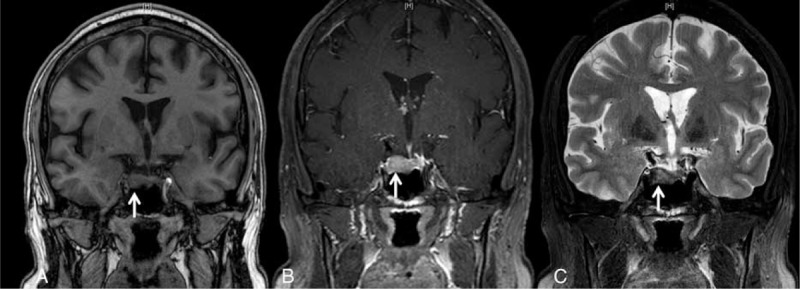
Pituitary magnetic resonance imaging. T1-weighted coronal (A), gadolinium-enhanced T1-weighted coronal (B), and T2-weighted coronal (C) views showing a 15 mm macroadenoma.

The clinical picture suggested a Cushing's syndrome: easy bruising, reddish purple striae (Fig. 1), proximal myopathy, facial fullness, as well as facial and truncal fat distribution. This was confirmed by blood tests showing severe hypokalemia (2.4 mmol/L), urinary-free cortisol (UFC) 14 times higher than the normal range (2016 nmol/L, N < 138), 8am ACTH level 7 times higher than the normal range (178 ng/L, N < 26), and by a failure of low-dose dexamethasone to suppress serum cortisol in a suppression test. Pituitary gadolinium-enhanced MRI revealed a macroadenoma measuring 15 mm in diameter (Fig. 2). Thus a final diagnosis was DCM complicating CD.

Pituitary surgery had to be postponed due to the severe heart failure.

Optimal pharmacological treatment of systolic heart failure was administered (beta-blocker, ACE inhibitor, diuretics including anti-aldosterone agent) and, as described in such severe conditions,^6,7^ 2 adrenolytic drugs (metyrapone and ketoconazole) were initiated: UFC was normalized within 2 months (Table 1).

**TABLE 1:**
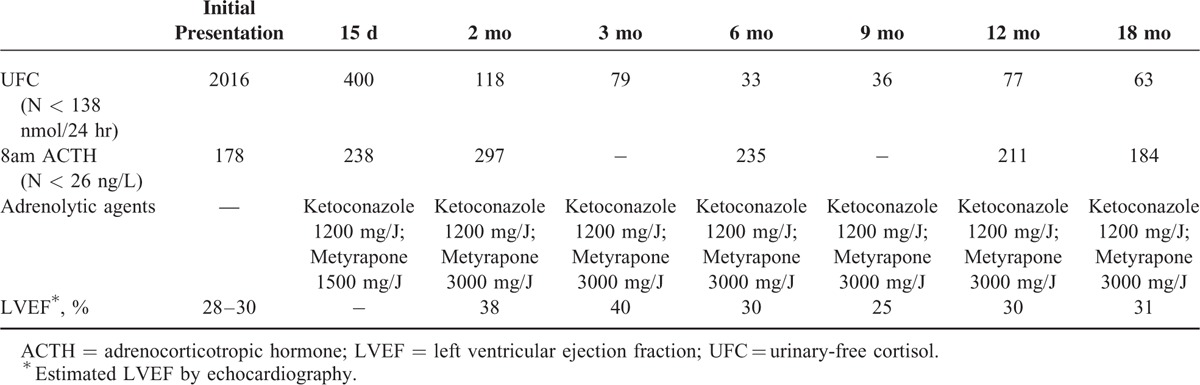
Evolution Under Anti-Cortisol Treatment of Urinary-Free Cortisol and LVEF as a Function of Time

**FIGURE 3:**
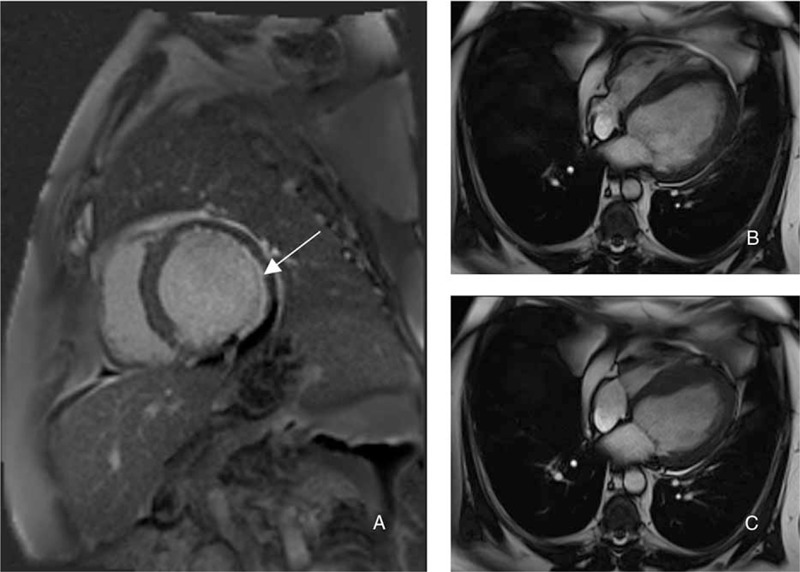
Cardiac magnetic resonance imaging. Late gadolinium enhanced image (A) showing a subendocardial posterolateral myocardial infarction. Cine-cardiac magnetic resonance acquisitions (diastole (B) and systole (C)) showing a severely impaired left ventricular function (LVEF estimated at 30%).

Unfortunately, a new episode of acute heart failure occurred two months after initial presentation. Transthoracic cardiac echography showed mitral insufficiency related to rupture of the mitral valve pillar. Percutaneous transluminal angioplasty and stenting (bare metal) was performed in the obtuse marginal artery. Another cardiac MRI then revealed an impaired lateral contractility (territory of the marginal branch) related this time to necrosis (Fig. 3). Infective endocarditis was ruled out (hemocultures were negative and no vegetation was visualized at transesophageal echocardiography).

In spite of optimal treatment and normalization of cortisol levels, no improvement was found in the LVEF 6 months after discovery of the Cushing's disease (Table 1).

Thus a single-chamber defibrillator was implanted for primary prevention, and percutaneous mitral valve repair (Mitraclip) was performed to treat the mitral regurgitation.

After 1 year, since LVEF remained at 30%, cardiac transplantation was discussed.

In view of the patient's heart failure, anesthesia for transphenoidal surgery was considered too dangerous; therefore, stereotaxic radiotherapy was performed to treat the CD 14 months after initial presentation. While awaiting the therapeutic effects of the pituitary irradiation, tolerance and efficacy of the 2 adrenolytic agents proved acceptable.

## DISCUSSION

Cardiovascular complications, including myocardial ischemia, left ventricular hypertrophy and cardiomegaly account in part for the higher mortality rate among patients with Cushing's syndrome.^8^ Some studies having examined the relationship between cardiac dysfunction and hypercortisolism, found that cardiac remodeling is independent of hypertension, and is probably due to a direct action of cortisol on myocardial tissue via glucocorticoid receptors.^2–4^

A recent cardiac MRI study showed that patients with Cushing's syndrome have lower left ventricular, right ventricular, and left atrial ejection fractions and increased left ventricular mass.^5^ Repetition of imaging after treatment revealed that these systolic dysfunctions and structural changes are reversible upon correction of hypercortisolism. None of the patients in this study had DCM.

Several cases with DCM related to hypercortisolism have been published^9–18^ (Table 2).

**TABLE 2:**
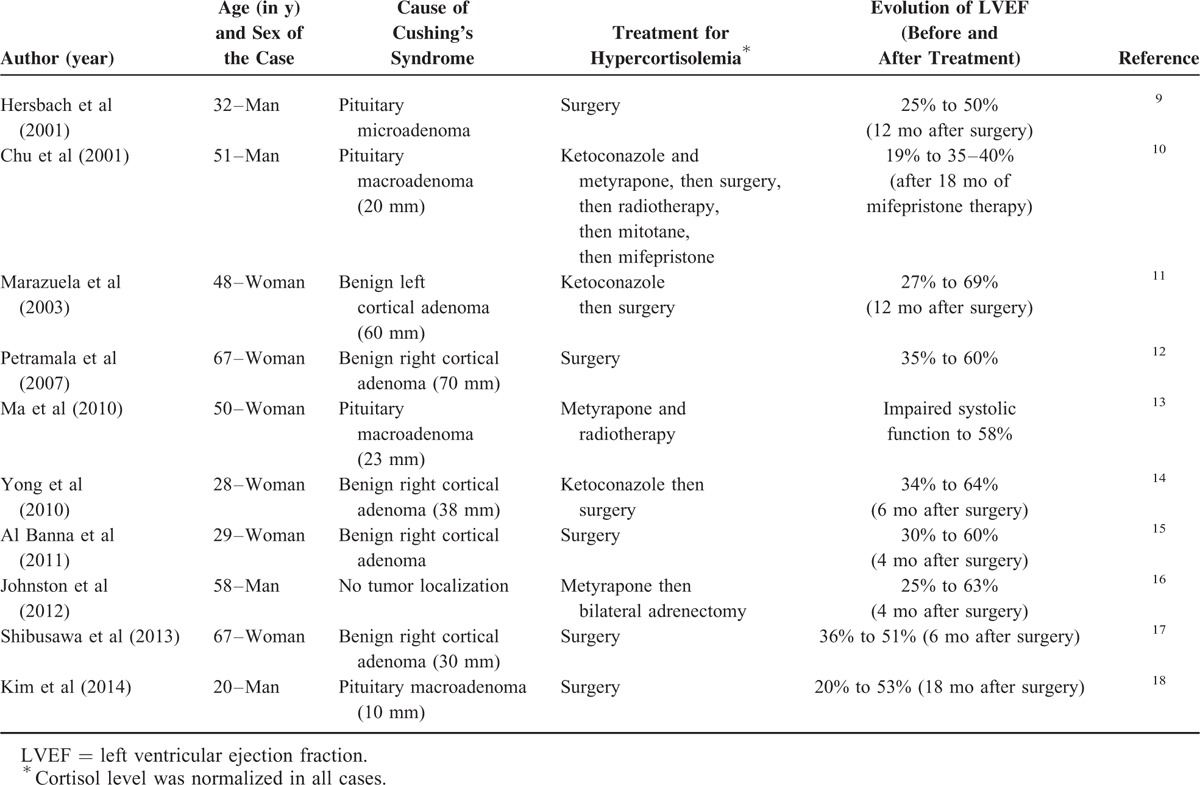
Case Reports of Dilated Cardiomyopathy Associated With Cushing's Syndrome

Interestingly, all these cases showed an improvement in LVEF after several months of cortisol normalization (median LVEF improvement of 30% [min: 15%; max: 42%]; in a median time of 9 months [min: 4; max: 18]). Cushing's syndrome was due to unilateral adrenal adenoma in 5 cases,^11,12,14,15,17^ ACTH-secreting adenoma in 4 cases,^9,10,13,18^ and of unknown cause in 1 case.^16^ LVEF improvement was noted whatever the initial etiology, with a median improvement of 30% (min: 15%; max: 42%) for adrenal adenomas, 25% (min: 15%; max: 33%) for pituitary adenomas, and 38% after bilateral adrenectomy in the subject for whom the cause was undetermined. In view of these observations, DCM related to hypercortisolism could be considered as “reversible.”

Here we have reported the first case showing no improvement of DCM in a context of Cushing's syndrome. At presentation the cardiomyopathy could only be attributed to hypercortisolism (usual other causes of DCM were eliminated, MRI initially showed no ischemic signs; however, unfortunately, several weeks later the patient suffered an acute coronary syndrome with rupture of the mitral pillar and objective signs of necrosis in the lateral territory (MRI). This combination of different causes of DCM (hypercortisolism, ischemia, and valvular defect) could explain the absence of improvement of LVEF in response to a eucortisolemic state.

Another possible explanation can be a publication bias due to the selective reporting of cases in which DCM had been reversed.

The standard treatment of CD is transphenoidal surgery with up to 65% remission rate for macroadenomas.^19^ For recurrent or residual disease, therapeutic possibilities are second transphenoidal surgery, radiotherapy, medical adrenolytic therapy (mainly comprising mitotane, metyrapone, and ketoconazole, and more rarely etomidate and mifepristone) or bilateral adrenalectomy in cases of uncontrolled cortisol levels.

Unfortunately for our patient, anesthesia for pituitary surgery was contraindicated due to very low LVEF. Combination of 2 adrenolytic agents (metyrapone, blocking the 11-beta-hydroxylase enzyme; and ketoconazole, blocking several steps in cortisol synthesis) normalized cortisol levels and tolerance was good. The observed efficacy is consistent with results from 2 studies in which this combination provided rapid control of severe hypercortisolism elicited by ACTH-dependent Cushing's syndrome^6^ (a combination of mitotane, metyrapone, and ketoconazole in this study) and by ectopic ACTH syndrome or adrenal carcinoma.^7^

Our patient was treated by external pituitary radiation, the therapeutic effects of which will be delayed.

In conclusion, we report here the case of a DCM as a presenting feature of CD, without improvement of LVEF after normalization of cortisol level (probably explained by subsequent associated ischemic lesions).
